# Citalopram associated with acute angle-closure glaucoma: case report

**DOI:** 10.1186/1471-2415-5-23

**Published:** 2005-10-04

**Authors:** Robert Croos, Srinivasa Thirumalai, Sabit Hassan, Jane Da Roza Davis

**Affiliations:** 1 Department of Psychiatry, Prospect Park Hospital, Reading, RG30 4EJ, UK; 2 Department of Ophthalmology, Royal Berkshire Hospital, Reading, RG1 5AN, UK; 3 Department of Psychiatry, Marlborough House Regional Secure Unit, Milton Keynes Hospital Site, Eaglestone, Standing Way, Milton Keynes, MK6 5NG, UK

## Abstract

**Background:**

Acute angle-closure glaucoma is a rare complication in patients receiving anti-depressant treatment. In the following case, we report the development of acute angle closure glaucoma in a patient who overdosed on Citalopram, an antidepressant, and discuss the possible etiological mechanisms for the condition.

**Case presentation:**

We report a 54 year old, Caucasian lady, with depression and alcohol dependence syndrome, who developed acute angle-closure glaucoma after an overdose of Citalopram, along with alcohol. She was treated with medications and had bilateral Yag laser iridotomies to correct the glaucoma and has made complete recovery. In this case, the underlying cause for glaucoma appears to be related to the ingestion of Citalopram.

**Conclusion:**

The patho-physiological basis for acute angle closure glaucoma in relation to antidepressant medications remains unclear. The authors suggest Citalopram may have a direct action on the Iris or Ciliary body muscle through serotonergic or anti-cholinergic mechanisms or both. This case highlights the importance of the awareness of the underlying risks, which may predispose an individual to develop acute angle-closure glaucoma, and reminds the clinicians the significance of history taking and examination of the eye before and after starting anti-depressants. This area needs to be further researched.

## Background

Depression is the most common psychological disorder in the world. The prevalence of unipolar depression is estimated to be between 3% and 13%, with as much as 20% of the world adult population experiencing at least some depressive symptoms at any given time [1]. Lifetime incidence of depression is estimated to be 20% to 55%. Approximately, 70% of moderately to severely depressed patients respond to anti-depressant therapy [2]. SSRI are increasingly the first line choice of anti-depressant because of their tolerable side-effect profile and low rate of lethality if taken in an overdose [3]. All SSRI are equally effective in treatment for depression [4].

Citalopram is an antidepressant of the selective serotonin reuptake inhibitor (SSRI) class. They act by producing a gradual increase in postsynaptic levels of serotonin (5-hydroxytryptamine, 5-HT) via desensitization of the feedback systems that controls the rate-limiting enzyme in 5-HT synthesis [5].

Serotonin (5-HT) receptors have been shown to be present in human eyes [6]. Furthermore, it is reported that Serotonin (5-HT) receptors are present at a higher concentration in mammalian ciliary body and cornea than in non-mammalian species [7]. Experimental studies have shown that topical application of serotonin increases the Intra-ocular pressure (IOP) in rabbit's eyes, and that 5-caboxamidotryptamine, a 5-HT 1a receptor agonist, is even more effective than 5-HT itself in elevating IOP [8]. Similarly, in a study of 20 consecutive depressed patients, following of a single dose of 20 mgs Fluoxetine it was shown to increase IOP by 4 mmHg [9]. In another study, Ketanserin, a compound with serotonergic blocking properties, reduced IOP in both animals and humans stressing the role of exerted by 5-HT on IOP [10].

Glaucoma is defined as a heterogeneous group of diseases that have in common a characteristic optic neuropathy and visual defects, for which elevated IOP is the primary risk factor [11]. There are approximately 67 million persons, worldwide, who suffer from glaucomatous disease of the eye [12]. These figures may not include the drug-induced glaucoma's because the precise information on the incidence of glaucoma as a result of local or systemic therapies is uncertain [13]. Angle-closure glaucoma is a disease with acute onset that occurs in 1 of 1000 Caucasians, about 1 in 100 Asians (especially mongoloids) and Hispanics, and 2–4 of 100 Inuit's (Eskimos) [13].

Risk factors for angle-closure glaucoma are narrow angle of anterior chamber, shallow anterior chamber depth, hyperopic, small eyes, positive family history of angle closure, elderly, female sex and use of medications that cause papillary dilatation and excitatory situations [14]. Drugs that cause or exacerbate angle-closure glaucoma include several classes of drugs including adrenergic agonists, cholinergics, anti-cholinergics, sulpha-based drugs, selective serotonin reuptake inhibitors, tricyclic and tetra cyclic antidepressants, anticoagulants and HI and H2 receptor antagonists, especially in people predisposed with narrow angles of anterior chamber. In some instances, bilateral involvement and blindness have occurred [11].

The patho-physiological mechanism of SSRI induced acute angle-closure glaucoma remains unclear, even though anti-cholinergic adverse effects or increased levels of serotonin, which cause partial papillary dilatation, have been implicated [11].

## Case presentation

We describe the case of a 54 year old, non-smoker, Caucasian woman, a computer programmer, who was admitted to the General Hospital in June 2003, following an episode of overdose with Citalopram and alcohol. At the time of her admission, she gave a history of depression and suicidal ideation for six months. She was not known to the local psychiatric service.

The patient was discovered by her twin sister soon after the overdose. Initially, she had disclosed to the medical doctor admitting her that she had taken approximately 14 tablets of 20 mgs of Citalopram along with 2 bottles of red wine. However, later on she informed us that she might have taken up to 30 tablets of 20 mgs Citalopram. The actual amount ingested remains unclear.

Soon after her admission, she complained of painful left eye with blurred vision, and was seen by the Ophthalmologist, who found that our patient had an intra-ocular pressure of 23 mmHg in the right eye and 60 mmHg of mercury in the left eye (Normal IOP-10–20 mm Hg), with left corneal edema, and fixed dilated pupils. She was not hypermetripic and had averaged sized eyes. She was noted to have shallow anterior chambers (central and peripheral depth not available) in both of her eyes. A diagnosis of left angle-closure glaucoma was made and medications were commenced to reduce the elevated IOP. Further investigations including routine blood investigations revealed no abnormalities. Her pulse rate and blood pressure were normal. She had a blood alcohol level of 85 mgs/dl (Less than 10 indicates safe levels and 50–100 indicates toxic levels). Her blood test did not reveal any detectable levels paracetamol or salicylates.

With regard to her background history, there was previous episode of overdose with paracetamol tablets in 1978. There was no history suggestive of physical illness, and specifically, no history of previous eye problems. There was no family history of eye related conditions. In December 2002, her general practitioner had diagnosed her to be suffering from depression with harmful misuse of alcohol, and commenced her on Citalopram 20 mgs daily. Later, she informed us that she had not taken any of this prescribed medication prior to her overdose and had only been taking Estrogens, given for hormone replacement (Prempak-C).

Approximately 48 hours after the overdose, on examination of her eyes, she was found to have left subhyaloid and retinal hemorrhages. After 72 hours, the visual acuity in the right eye was 6/9 and hand movements in the left eye. The intraocular pressures were reasonably controlled and she had bilateral Yag laser iridotomies. Subsequently, she was discharged to the local psychiatric unit as she continued to express suicidal thoughts and was low in mood. Apart from receiving treatment for her eye problems at the general hospital, she was commenced on a reducing regime of chlordiazepoxide for her alcohol dependence. On discharge from the general hospital, she was advised to continue on Timolol, pilocarpine and dexamethasone eye drops for further 14 days.

She remained free of anti-depressants and her mood improved gradually in the absence of alcohol. After 14 days, her visual acuity was noted to be 6/18 in the left eye, which improved to 6/12 on pinhole. Her ocular condition was noted to be stable. Her ocular pressures were 14 mmHg in the right eye and 15 mmHg in the left eye, but still had retinal hemorrhages on the left eye, with a clearing vitreous hemorrhage.

She was followed-up by the Ophthalmologists and discharged from the psychiatric hospital after 4 weeks with further community support.

In August 2004, her right eye visual acuity was noted to be 6/9, which improved to 6/6 with pinhole and her left eye vision was 6/24, which did not improve with pinhole. She had a left afferent papillary deficit. The pre-retinal and retinal hemorrhages had cleared, but she still had some residual vitreous hemorrhage. Her right eye Intra-ocular pressure was 18 mmHg and left was 19 mmHg without any treatment. She is reviewed by the local eye clinic every six months.

## Discussion

Tricyclic antidepressants, such as, amitriptyline and imipramine have been associated with acute angle closure glaucoma [[15] &[16]]. Although there are eight reports of glaucoma and amitriptyline, there is only one reported case of association of amitriptyline with acute angle closure glaucoma and this had taken place following an episode of overdose [15]. In another study, four patients with narrow angles suffered from acute angle closure glaucoma after routinely prescribed doses of imipramine [16]. Bilateral acute angle closure glaucoma has been reported in a hypermetripic patient on venlafaxine and chlorpromazine, suggesting, perhaps, this occurred by the hepatic inhibition of chlorpromazine metabolism of venlafaxine, increasing anticholinnergic activity, or by a direct effect of venlafaxine on the eye, unrelated to mydriasis [17]. The manufacturers of venlafaxine (Wyeth Laboratories) report glaucoma as a rare adverse event. There has been one previous report of increased intraocular pressure in two patients with known narrow angle glaucoma who began taking venlafaxine [18].

Bilateral angle closure glaucoma and visual loss was precipitated by maprotiline and alprazolam in a 71 years old lady with a history of depression, who had previously complained of ocular pain and blurred vision [19]. Fluoxetine, Paroxetine and fluoxamine have been associated with angle closure glaucoma [[11] &[20]]. Voluntary reporting of suspected adverse events with Fluoxetine has identified a total of 63 cases of glaucoma in an estimated patient population of 21 million in 1998 [21]. The manufacturers of Paroxetine are aware of four cases of acute angle closure glaucoma, and one of raised IOP in a UK patient population of over a million in 1998 [21]. The manufacturers of Citalopram are aware of 15 reports of glaucoma, but causality has not been assigned in these cases and there is no published literature concerning glaucoma as a recognized adverse effect after overdose with Citalopram (Lundbeck Ltd, personal communication).

To our knowledge, this is the first report of acute, unilateral, angle closure glaucoma in a patient following an overdose of Citalopram antidepressant. In our case, there was a clear temporal link between Citalopram overdose and the development of acute angle closure glaucoma. It may be that the onset of glaucoma may be due to the rapid rise in blood concentration of the drug after the overdose (serum concentration of Citalopram was not measured in our case). It is also possible that our patient was predisposed to develop glaucoma due some other unknown inherent factor. Although, it would have been ideal to reintroduce our patient to Citalopram in order to demonstrate causality, especially as our patient had surgical intervention in both of her eyes, we decided this was not in the best interest our patient as the risk of another episode of glaucoma would be unacceptable.

If there is a causal relationship, it could be proposed that Citalopram may directly act on the iris or ciliary body muscle through serotonergic or cholinergic mechanisms or both.

We think our case demonstrates that the need to prescribe Citalopram and other SSRI with caution, especially in older female patients, anatomically predisposed individuals, glaucoma patients and those with a family history of glaucoma. We would like to suggest that careful consideration should be given to include history taking and fundoscopic examination before and after starting SSRI in depressed patients.

We think this area merits further investigation and colleagues should continue to report cases of glaucoma or raised IOP to relevant Drugs safety committees.

## Abbreviations

SSRI – Selective serotonin reuptake inhibitor

5-HT-5-Hydroxytriptyamine

IOP – Intra ocular pressure

## Competing interests

RC – None

SH – None

JDRD – None

ST-Has been sponsored to attend meetings by Astra Zeneca, Sanofi-synthelabo, Eli-Lilly, Pfizer, Novartis and Wyeth pharmaceuticals.

## Authors' contributions

RC-participated in information gathering, literature search, data analysis, drafting and co-ordination of the case report. SH-Participated by managing the patients ophthalmologic problems and helped to draft the manuscript. JDRD-Involvement in the psychiatric management, co-ordination of the work and co-wrote the Manuscript. ST-conceived the idea, participated in information gathering, literature search, data analysis, and psychiatric management of the case and drafting the final manuscript. All authors read and approved the final manuscript.

## Pre-publication history

The pre-publication history for this paper can be accessed here:


